# Surgical Management of Cardiac Hydatid Cyst and the Residual Intramural Ectocyst

**DOI:** 10.7759/cureus.9829

**Published:** 2020-08-18

**Authors:** Firas F Ibrahim, David Rubay, Slee Yi, Zuhair Barqawi, Ali N Abed

**Affiliations:** 1 Cardiac Surgery, Iraqi Center of Heart Diseases, Baghdad, IRQ; 2 Trauma and Surgical Critical Care, University of Florida College of Medicine, Gainesville, USA; 3 Surgery, Charles E. Schmidt College of Medicine, Florida Atlantic University, Boca Raton, USA; 4 Surgery, University of Colorado School of Medicine, Aurora, USA; 5 Cardiac Surgery, Iraqi Center for Heart Diseases/Medical City Teaching Complex, Baghdad, IRQ

**Keywords:** cyst hydatid, echinococcus granulosus, enzyme-linked immunosorbent assay (elisa)

## Abstract

The cardiac hydatid cyst (HC) is a rare pathology and mostly is endemic in livestock raising countries. Patients do not have a specific presentation so it is mainly a diagnosis based on imaging. Finding HC anywhere in the body warrants looking for another hydatid in other organs. This is a case report of a young male who presented with nonspecific symptoms and during diagnostic workup, it happened that he has combined hepatic and cardiac HCs. The cardiac cyst was located intramurally in the interventricular septum and expanding down mostly to the left side of the diaphragmatic surface of the heart and partly crossing intramurally to the diaphragmatic surface of the right ventricle. Emergency open-heart surgery was performed; the endocyst was removed while intramural ectocyst was drained to prevent potential future residual space.

## Introduction

Hydatid cyst (HC) is a human parasitic disease caused by the cestode tapeworm Echinococcus granulosus, which infests the guts of dogs as its definitive host while human beings are accidental hosts and are infested by the ingestion of ova in vegetables or water contaminated with dog feces [1]. HC was described back in the 17th century and it is thought that it came from Iceland and brought to Europe by dogs accompanying whaleboats in the 18th century [2]. It is found that HC is endemic in livestock raising countries [3]. HC is commonly located in the liver (>65% of cases) and the lungs (25%) [1]. However, even in countries where hydatid disease is endemic, only sporadic isolated cases of cardiac involvement (0.5%-2% of all cases) have been reported [2, 4-6]. Cardiac echinococcosis was first described in 1836 and the first successful operation for cardiac echinococcosis was performed by Long in 1932 [1]. When echinococcosis is diagnosed anywhere in the body then the treatment of choice, even for asymptomatic cases, is surgical removal due to the risk of cystic rupture into body cavities or circulation leading to anaphylactic shock or dissemination via circulation to other organs leading to what is called malignant hydatidosis [1, 4, 7]. In nonoperable cases then long-term therapy with albendazole is the option [8].

The distribution of echinococcosis in the heart depends on the blood supply to that part of the heart. The coronary circulation is the main pathway by which the parasitic larvae reach the heart and because of rich coronary blood supply, the left ventricular wall is the most common cardiac location (60%), followed by the right ventricle (10%), pericardium (7%), left atrium (6%-8%), and right atrium (3%-4%) [3, 9]. Cardiac cysts are mostly asymptomatic but they may present with serious and fatal complications such as cardiac tamponade, embolization, chest pain, valvular regurgitation, and very rarely, with arrhythmias [10]. Cystic echinococcosis is one of the few parasitic infections in which the basis for laboratory diagnosis is primarily serologic, where indirect hemagglutination test and enzyme-linked immunosorbent assay (ELISA) are the most widely used methods for detection of anti-Echinococcus antibodies (immunoglobulin G [IgG]) [11]. HC is composed of host tissue forming ectocyst then laminated layer, germinal layer, brood capsules containing protoscolices which can develop to secondary HC in the intermediate host viscera in case of rupture of the cyst [12].

Transthoracic echocardiography is the elementary imaging technique but it carries several well-described limitations, including operator dependence, restricted field of view in patients with large body habitus, and limited views of the left ventricular apex and the right heart chambers. CCT provides useful information in detecting specific findings and performs better than transthoracic echocardiography by distinguishing cysts from solid tumors such as myxomas or fibromas through using density measurements and analysis of enhancement after IV contrast injection [13].

## Case presentation

**Figure 1:**
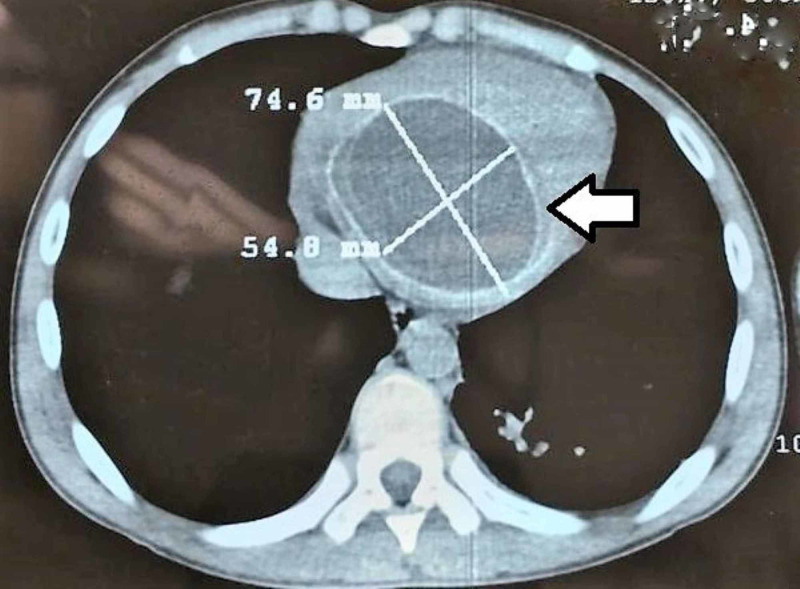
CT scan of the chest showing the intramyocardial cyst (axial view).

**Figure 2:**
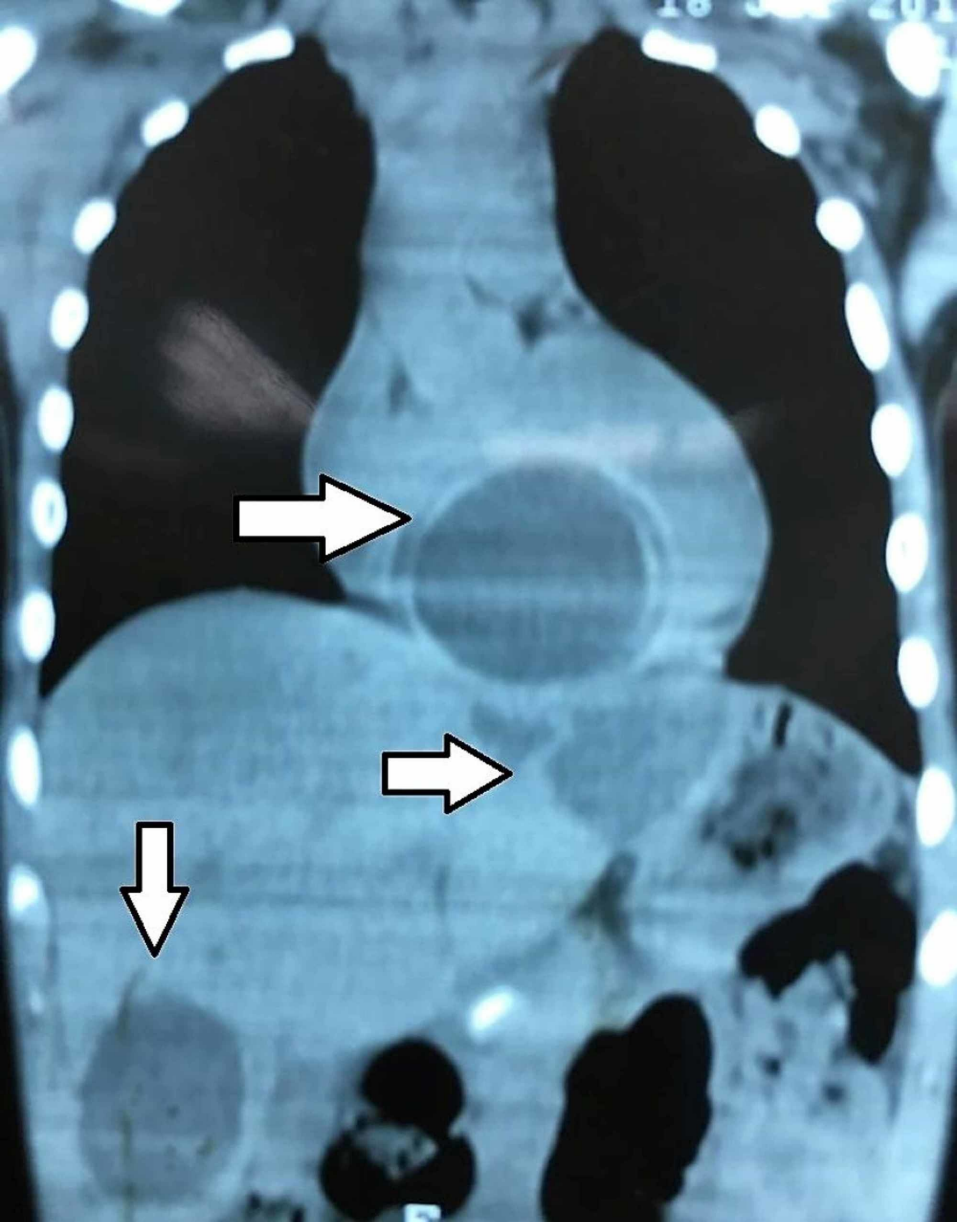
CT scan of the chest and abdomen showing cysts within the liver and heart (coronal view).

**Figure 3:**
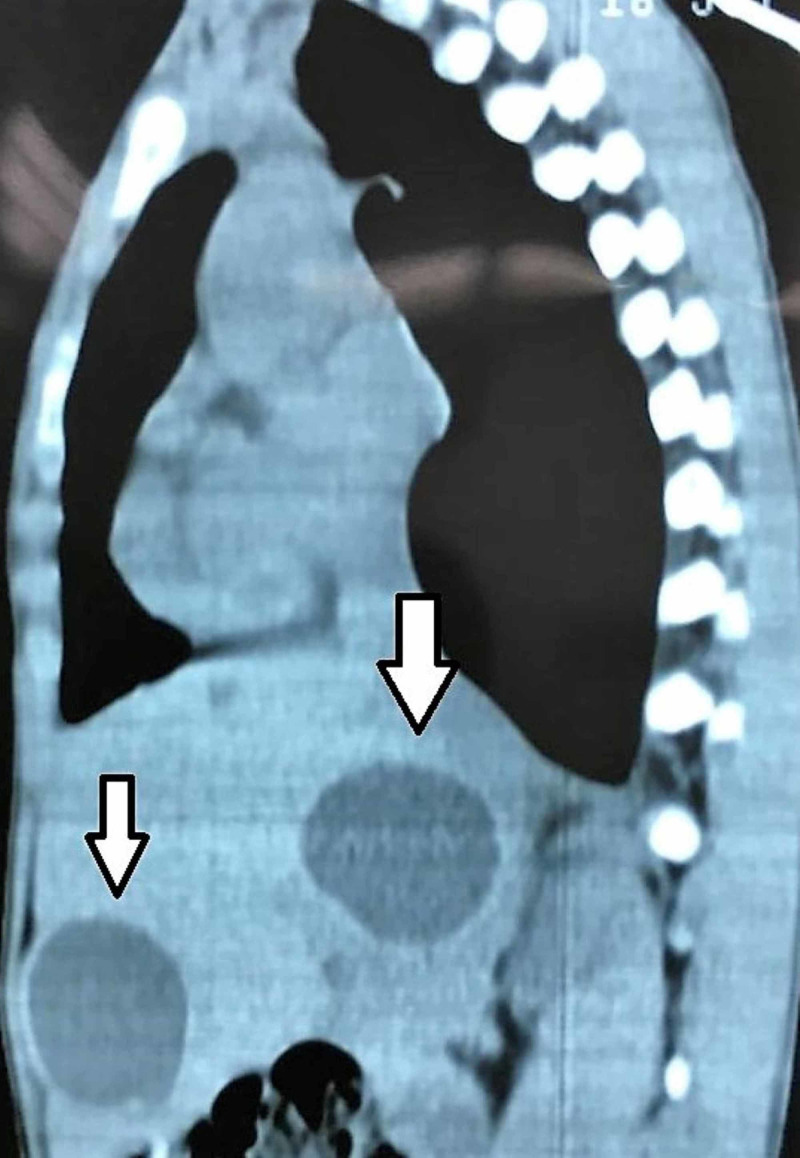
CT scan of the abdomen and chest showing cysts in the liver (sagittal view).

We are presenting a case of a 15-year-old male from a rural area living on a farm and raising livestock. He presented with six months history of easy fatigability, shortness of breath, and dull right upper abdominal pain. Physical examination revealed that the patient had tachycardia at rest and hepatomegaly and no other significant findings. Chest X-ray (CXR) showed clear lung fields and mildly enlarged heart. Ultrasound examination of the abdomen revealed few cysts in the liver without any associated lymphadenopathy in the coeliac group of lymph nodes. As part of the workup, the patient had a CT scan of the abdomen, chest, and brain. The CT showed one cyst in the heart, four cysts in the liver, but no cysts in the lungs or the brain (Figures 1-3).

**Figure 4:**
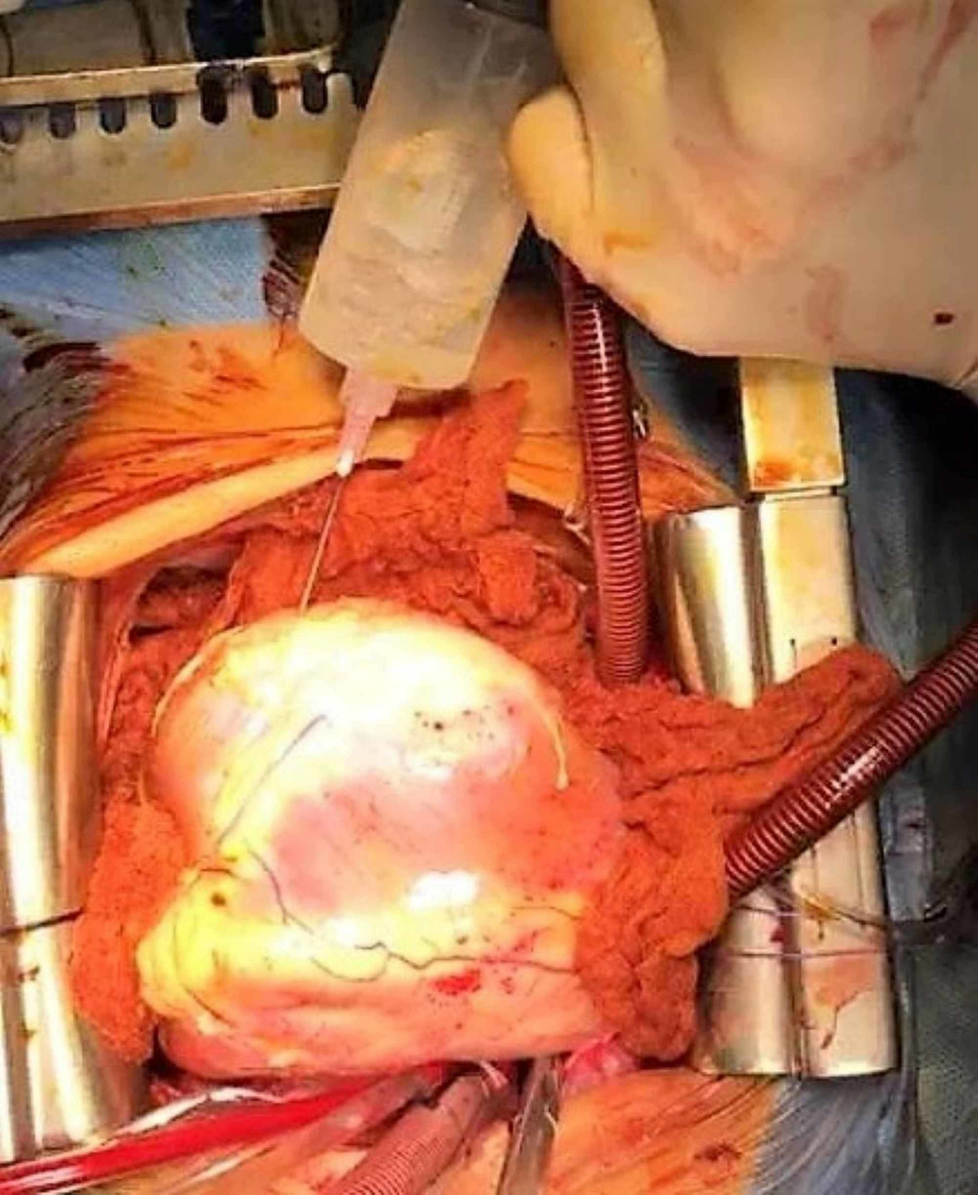
Picture demonstrating the aspiration of the cyst.

The cystic nature of the lesions and their locations in multiple organs made us think of the HC as the pathology. The serology test for HC using the ELISA test came positive, which helped to confirm the diagnosis. The patient was scheduled for urgent open-heart surgery, fearing the risk of potential rupture of the cyst and the grave consequences which come with that. The patient underwent conventional median sternotomy and aortocaval cannulation with aortic cross-clamp and cardioplegic heart arrest. CT images helped to locate the cardia cyst and its relation to the cardiac chambers. The location of the cyst was confirmed by direct examination of the heart when it was arrested. The cyst was totally located intramurally in the interventricular septum and expanding down mostly to the left side of the diaphragmatic surface of the heart and partly crossing intramurally to the diaphragmatic surface of the right ventricle making a visible and palpable soft cystic bulge. The myocardium overlying the cyst on the left side of the posterior descending coronary artery on the diaphragmatic surface of the heart was fibrosed, making part of the ectocyst wall. This was the most epicardially prominent part of the cyst and was chosen as the site of aspiration and later incision to evacuate and remove the cyst. Initially, a 50 mL syringe was used to aspirate the cyst and make it less tense to avoid rupturing it during incision (Figure 4).

**Figure 5:**
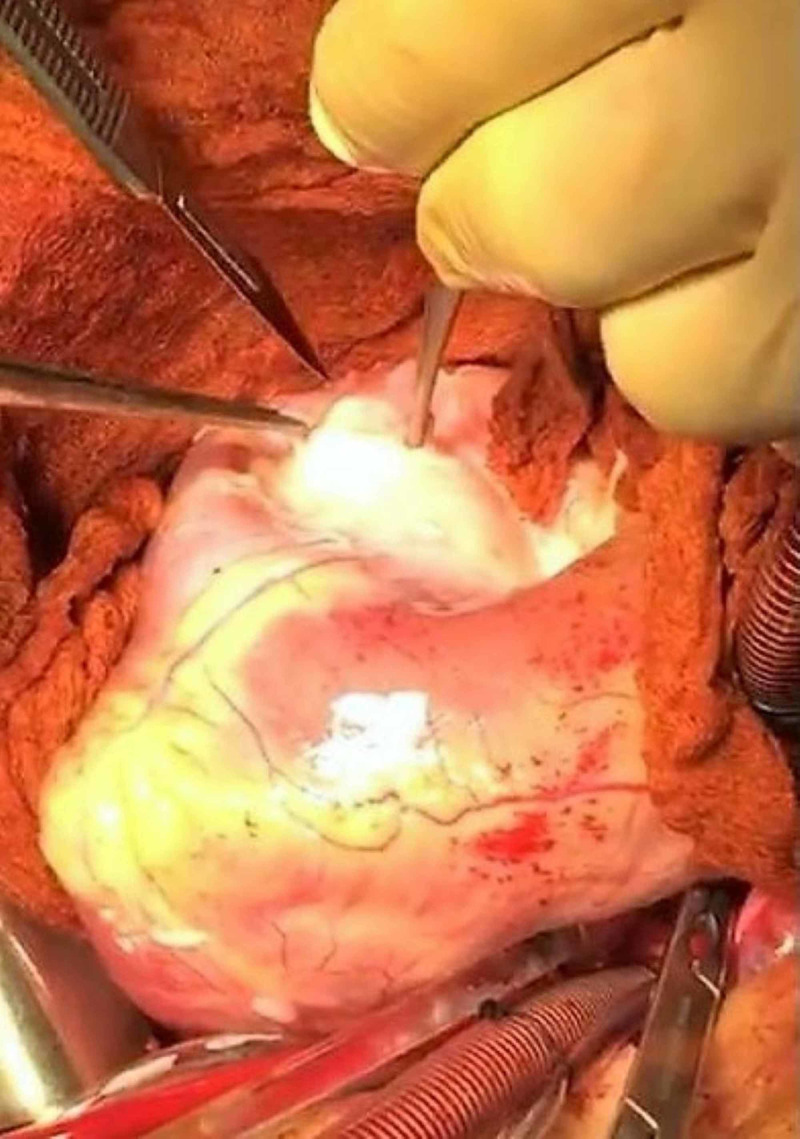
Surgical incision of the fibrotic ectocyst.

Around 200 mL of clear fluid was aspirated, and the bulge disappeared. After the evacuation of most of the fluid, a small surgical incision of the fibrotic ectocyst was performed from the same site of aspiration (Figure 5).

**Figure 6:**
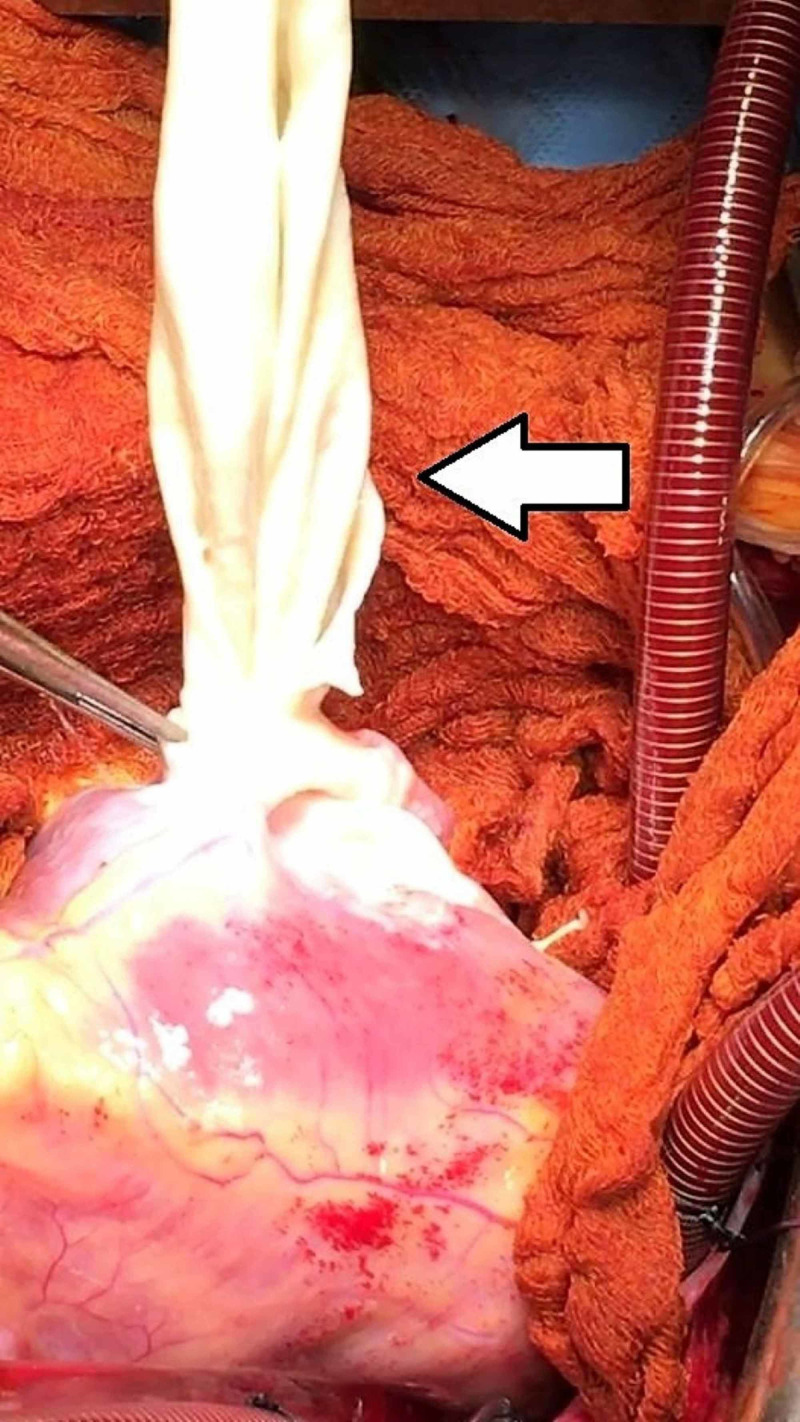
Extraction of the cyst out of the cavity using sponge forceps.

A surgical discard sucker tip was introduced via the incision, and some residual fluid was aspirated. The incision was enlarged to admit forceps to take the cyst out. There was a residual cavity left behind intramurally caused by the cyst. The residual cavity was inspected to make sure that no part of the cyst was left behind, and the wall was intact, and no communication with cardiac chambers was noticed as no blood was ever seen to collect. The cyst and the fluid were sent to the histopathological and cytological exam (Figure 6).

The cavity left draining to the base of the heart without closure of the surgical incision, hoping that the normal filling of the ventricular chambers will eventually lead to the collapse of the residual cavity and prevent any future collection. The patient weaned successfully from the heart and lung machine, and a mediastinal drain was left on the diaphragmatic surface of the heart and another on the anterior surface of the heart. The patient was then discharged to the cardiac intensive care unit (CICU). After a few hours the patient was extubated from the ventilator without any pharmacological support. After 24 hours, CXR and echocardiography were repeated, and both were normal. The patient was discharged home after six days very stable and was referred to general surgery to deal with the liver cysts in the future.

## Discussion

Human acquires hydatid disease through ingesting food or milk or water contaminated by dog feces containing the ova of the parasite [6, 11]. Cardiac HC is rare (0.5%-2%) compared to the liver (in 50%-70% of cases) [1, 3]. And the involvement of the left ventricle and the interventricular septum is more common than other chambers of the heart [1, 5, 14]. There are no typical clinical signs and symptoms to draw attention to the possibility of the diagnosis, and many are asymptomatic and discovered accidentally [3]. Our patient was not an exception regarding his typical clinical picture and the accidental discovery of the heart cyst. What should be emphasized here is that once you find one HC anywhere in the body, then search for others in other organs because of the systemic nature of the pathology [3]. It is recommended that HC should be considered as a differential of all cystic masses in all anatomical locations, including unusual sites like myocardium, especially in areas endemic with the disease [15]. The use of CT was very helpful in the precise localization of the cysts and screening other vital organs like the brain, lungs, and heart [13]. Late on echocardiography was used to delineate further the cyst and its relation to cardiac chambers and structures [8]. What is necessary for us while planning the surgery was to avoid unnecessary extensive cutting in viable myocardium and avoid vital structures like a valve, coronaries, and conductive system. And we think that selecting the epicardial surface of the heart will ensure no spill of the hydatid fluid inside the cardiac cavity to avoid secondary HC and drainage of the cyst to the pericardial cavity [12]. The cyst had already created a bulge on the diaphragmatic surface of the heart leading to some thickening of the myocardium in the area as part of its ectocyst. Choosing that part ensured easier access to the cyst without damaging vital structures within the heart. Moreover, it provided the drainage of the residual cavity both by the expanding ventricles and the dependent location of the incision. Despite that, there was no clinical or radiological feature of communication between the cyst and the cardiac chambers. Yet, it was necessary during surgery to check the interior of the residual cavity in order not to miss any communication between the cardiac chambers and the cavity, which then would require repair.

## Conclusions

Cardiac HC is a rare and very serious and life-threatening cardiac pathology. It does not have a typical clinical presentation, where patients may present with unexplained arrhythmia and shortness of breath. Usually, it is located intramurally and mostly in the left ventricular wall and can be single or multiple cysts. Physicians should keep a high index of suspicion among patients in areas endemic with the disease. Because of the potentially systemic nature of the disease, finding a HC anywhere in the body should trigger a search in other parts. Surgical management of cardiac HC requires urgent surgical removal of the endocyst using epicardial incision to avoid intracardiac contamination with the infective HC fluid and to ensure drainage of the residual ectocyst to the pericardial space and providing no communication between the cardiac chambers and the residual cavity.
